# The Field of Science

**Published:** 1894-06-02

**Authors:** 


### The Field of Science.

A laboratory for the preparation of vaccines, bearing M. Pasteur's name, and on the great scientist's methods, will be shortly established at Würtemberg by the Veterinary Section of the local Academy of Medicine.

---

Three research scholarships, each of the value of £250 and open to British subjects, have been instituted by the Grocers' Company "as an encouragement to the making of exact researches into the causes and prevention of important diseases."

---

An American contemporary gives us an astonishing instance of a city which appears to cling to what one might almost term pre-historically sanitary conditions, or insanitary conditions, rather. This is in the case of the town of Pittsburg. It appears that all efforts to improve the state of the streets is steadfastly resisted on the part of the inhabitants. Refuse is removed by private companies, if at all; the poorer classes cannot and do not remove it, and it simply accumulates. Alleghany City has the highest death-rate in the country, we learn, from typhoid fever, and Pittsburg is a close second. Lately, the Women's Health Protective Association of Pittsburg attempted to improve matters by organizing a municipal system for the removal of garbage, but without success.

---

The Pittsburg *Medical Review* editorially states that one of Alleghany's,philanthropic select Councilmen proposes to present a drinking fountain to the city, and the following inscription has been humorously suggested for the same: "Erected to the memory of 161 citizens who drank of this water and died of typhoid fever during the year 1893. This water is warranted to be drawn from the Alleghany River, at a point where the discharges of eighteen sewers of Pittsburg are mingled with the stream, and that each drop contains on an average 200 bacteria."

---

It has lately been pointed out that man, after all, is not the only animal who indulges in stimulants. Certain animals also indulge themselves in this manner with fatal results, the vehicle of intoxication in the case of the lower order of beings being a creeping vetch called the "Loco Plant." This is an inhabitant of the "Texan Panhandle," and is a source of serious danger to horses and cattle. To them it has all the allurements which are possessed by absinthe and gin for beings of another grade, only the results of the vetch are more definitely fatal, even when taken in moderate rmeasures. Dr. Carhart, whose studies in this direction throw much light on the consequences of loco eating, describes the most prominent features of the poisoning on the part of the victim, as the entire loss of muscular co-ordination. Animals who have tasted of it are liable to fall over backwards, their brains being affected as well as their spinal cords. They leap heights in their frenzy, and dash down precipices. No rider of a horse who has been thus intoxicated is in a safe position.

The appointment of Dr. Mallasez, member of the Academy of Medicine, as successor to M. Charcot, seems to have given universal satisfaction in Paris. M. Charcot's successor is described as being a very prolific writer; no fewer than 213 publications are credited to come from his pen.

---

The Emperor of Austria has just made a graceful recognition of the important services rendered to science by the geographical survey of Inda, by the presentation of gold medals to two senior members of the survey, Dr. W. King and Mr. C. L. Griesbach.

---

Arrangements were made in Amsterdam for the celebration of Lavoisier's Centenary last month. A commission, nominated by the physical section of the Amsterdam Society for the Advancement of Physics and Medicine, was entrusted with the carrying out of the same. The commemorative address was delivered by Dr. Van Deventer who described the apparatus of the Dutch physicist, Van Marum, by means of which he repeated Lavoisier's experiments on combustion. The original apparatus has since been improved on by Van Marum, and is contained in the museum of Teyler's Society at Harlem.

---

The rise in the price of spirits will be felt in an indirect manner by medical men. A proportionate rise must also be looked for in phaımaceutical preparations into which spirits enter. This has already been pointed out elsewhere. We quote an extract from the resolution lately passed by the House of Commons: "And the duties of Customs on the articles thereinafter mentioned being articles of which spirits are a part or ingredient, shall be proportionately increased, and shall be as follows: Chloral hydrate, 1s. 4d. per pound; chloroform, 3s. 3d. per pound; collodion, £1 3s. per gallon; ether ascetic, 1s. 11d. per pound; ether butyric, 16s. 5d. per gallon; ether sulphuric, £1 7s. 5d. per gallon; iodide of ethyl, 14s. 3d. per gallon.

---

The official report lately issued of the Institute Pasteur shows, as has been pointed out, that though a similar establishment is opposed in this country, yet that we are not loth in availing ourselves of the benefits of anti-rabic treatment in Paris. Of the 1,648 persons who were treated at the Institute during the past year only four cases terminated fatally. The mortality table only refers to deaths from hydrophobia which occurred after the lapse of fifteen days from the date of the last inoculation, such deaths being regarded as taking place in spite of treatment. Deaths occurring within this prescribed period are excluded. Last year two such took place, which are excluded from those registered. The following table shows the nationality of the patients:—

| | | | |
|---|---|---|---|
| Germany ... | ... 2 | Greece ... | ... 35 |
| England ... | ... 23 | Holland ... | ... 9 |
| Austria ... | ... 1 | India ... | ... 14 |
| Belgium ... | ... 22 | Morocco ... | ... 1 |
| Brazil ... | ... 1 | Portugal ... | ... 6 |
| Egypt ... | ... 18 | Russia ... | ... 1 |
| Spain ... | ... 43 | Switzerland | ... 9 |
| United States | ... 1 | Turkey ... | ... 2 |

